# Application of deep learning in detecting neurological disorders from magnetic resonance images: a survey on the detection of Alzheimer’s disease, Parkinson’s disease and schizophrenia

**DOI:** 10.1186/s40708-020-00112-2

**Published:** 2020-10-09

**Authors:** Manan Binth Taj Noor, Nusrat Zerin Zenia, M Shamim Kaiser, Shamim Al Mamun, Mufti Mahmud

**Affiliations:** 1 Institute of Information Technology, Jahangirnagar University, Savar, 1342 Dhaka, Bangladesh; 2 Department of Computing & Technology, Nottingham Trent University, NG11 8NS Nottingham, UK

**Keywords:** Machine learning, Alzheimer’s disease, Parkinson’s disease, Schizophrenia, Neuroimaging

## Abstract

Neuroimaging, in particular magnetic resonance imaging (MRI), has been playing an important role in understanding brain functionalities and its disorders during the last couple of decades. These cutting-edge MRI scans, supported by high-performance computational tools and novel ML techniques, have opened up possibilities to unprecedentedly identify neurological disorders. However, similarities in disease phenotypes make it very difficult to detect such disorders accurately from the acquired neuroimaging data. This article critically examines and compares performances of the existing deep learning (DL)-based methods to detect neurological disorders—focusing on Alzheimer’s disease, Parkinson’s disease and schizophrenia—from MRI data acquired using different modalities including functional and structural MRI. The comparative performance analysis of various DL architectures across different disorders and imaging modalities suggests that the Convolutional Neural Network outperforms other methods in detecting neurological disorders. Towards the end, a number of current research challenges are indicated and some possible future research directions are provided.

## Introduction

Alzheimer’s disease (AD), Parkinson’s disease (PD) and schizophrenia (SZ) are three most common neurological disorders (NLD) which are characterized by the disruption of regular operations of brain functions [1–3]. A patient with either of these three NLD puts a heavy burden on the family as well as the health system. It is therefore imperative to detect these disorders at the earliest stage possible so that their progression can be slowed down, if not fully stopped [4, 5]. Towards this aim, a number of different neuroimaging techniques (such as magnetic resonance imaging (MRI), computed tomography (CT) and positron emission tomography (PET)) and deep learning (DL)-based analysis methods have been developed to classify these disorders for early detection [3, 6–8], and to devise appropriate treatment strategies [9–11].

**Fig. 1:**
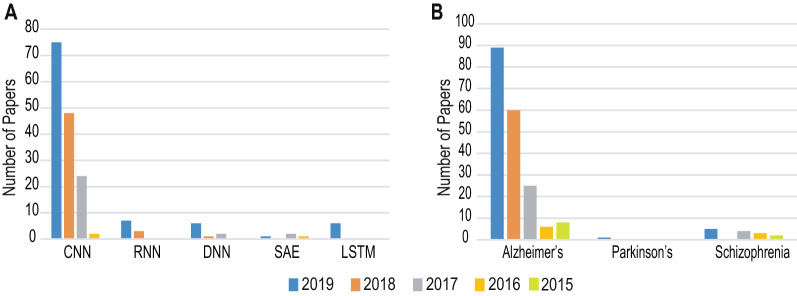
Peer-reviewed research results published during the last 5 years reporting the usage of DL in detecting NLD from MRI data. The Scopus database (https://www.scopus.com/) was searched with search-strings containing keywords “Deep learning” and “MRI” in conjunction with each of the NLD (“Alzheimer’s”, “Parkinson’s”, and “schizophrenia”) and the obtained results were categorized basing on the DL architectures (**a**) and diseases (**b**). A. In the literature the CNN has been reported much more frequently in comparison to the RNN, LSTM, DNN, and AE. B. The main effort appears to cluster around AD in comparison to PD and SZ

Over the last decade machine learning (ML) has been successfully applied to biological data mining [12, 13], image analysis [14], financial forecasting [15], anomaly detection [16, 17], disease detection [18, 19], natural language processing [20, 21] and strategic game playing [22]. In particular, the success of DL algorithms in computer vision, researchers of neuroimaging have also strived to use DL-based approaches for the detection of these NLD from MRI scans [3, 23–25]. Also, it is noteworthy that multimodal approaches including data fusion has also been used in diverse fields including diagnosis of neurological disorders [26] as well as providing personalized services [27]. As shown in Fig. 1, the number of research findings reported in peer-reviewed avenues have been increasing every year. Out of the large number of DL architectures, researchers have been mainly relying on Convolutional Neural Network (CNN)-based approaches for detecting these NLD from MRI data in comparison to other architectures such as Recurrent Neural Network (RNN) and Long–Short Term Memory (LSTM), Deep Neural Network (DNN), and Autoencoder (AE) (see Fig. 1a). Additionally, the detection of AD has attracted much more attention in comparison to PD or SZ in the published literature over the years from 2015 to 2019 (see Fig. 1b).

Due to the increasing interest in this field and the surging number of reported approaches to analyze the MRI scans, it is a timely demand to summarize the existing literature to facilitate the selection of an appropriate technique for a given task and dataset. There exist some reviews summarizing the advances from different perspectives. One among them aims to synthesize the applications of ML and big data to study mental health [6]. Various ML-based tasks have been explored on connectome data from MRI which aims to better diagnose neurological disorders [28]. In [29] authors have investigated the application of DL to better understand and diagnose PD. A detailed survey on DL applied to the analysis of various medical image such as neuro, pulmonary, pathology, etc., has been conducted in [30]. However, the preprocessing and data selection are not discussed clearly in any of the available reviews. To mitigate this gap, the objective of this work is to put forth an overview of the DL’s application in detecting NLD (i.e., AD, PD and SZ) from MRI scans along with popular open-access datasets and pre-processing methods. Therefore, the main contributions of this work are:

- A succinct introduction with appropriate sign-posting to different DL architectures and pre-processing techniques used in detecting abnormalities from the MRI scans. This will set the scene for a new entrant to the field and serve as a future reference.
- A detailed account on the existing studies which reported the application of DL on MRI scans for the detection and classification of AD, PD and SZ. To the best of our knowledge, this is the first attempt in reviewing the DL-based classification approaches of these three NLD variants from MRI.
- A full report of the popular open-access datasets along with their sources and detailed information about the participants (e.g., number of subjects, age, gender, etc.), and MRI scan modality. This will facilitate the validation and comparison of a new method’s performance using open-access benchmark datasets.
- A focused discussion on the current research challenges and some future research directions to guide the new entrants towards impactful development.

The subsequent sections of the paper are organized as: Sect. 2 succinctly describes various DL architectures used in analyzing MRI scans to detect AD, PD and SZ. Section 3 discusses the popular pre-processing techniques while sect. 4 provides the detailed account on the detection of AD, PD, and SZ. Section 5 presents the existing open-access datasets available for exploration. Section 6 includes performance analysis of the reviewed methods and sect. 7 offers challenges and future perspective with sect. 8 concludes the work.

## Overview of deep learning techniques

**Fig. 2:**
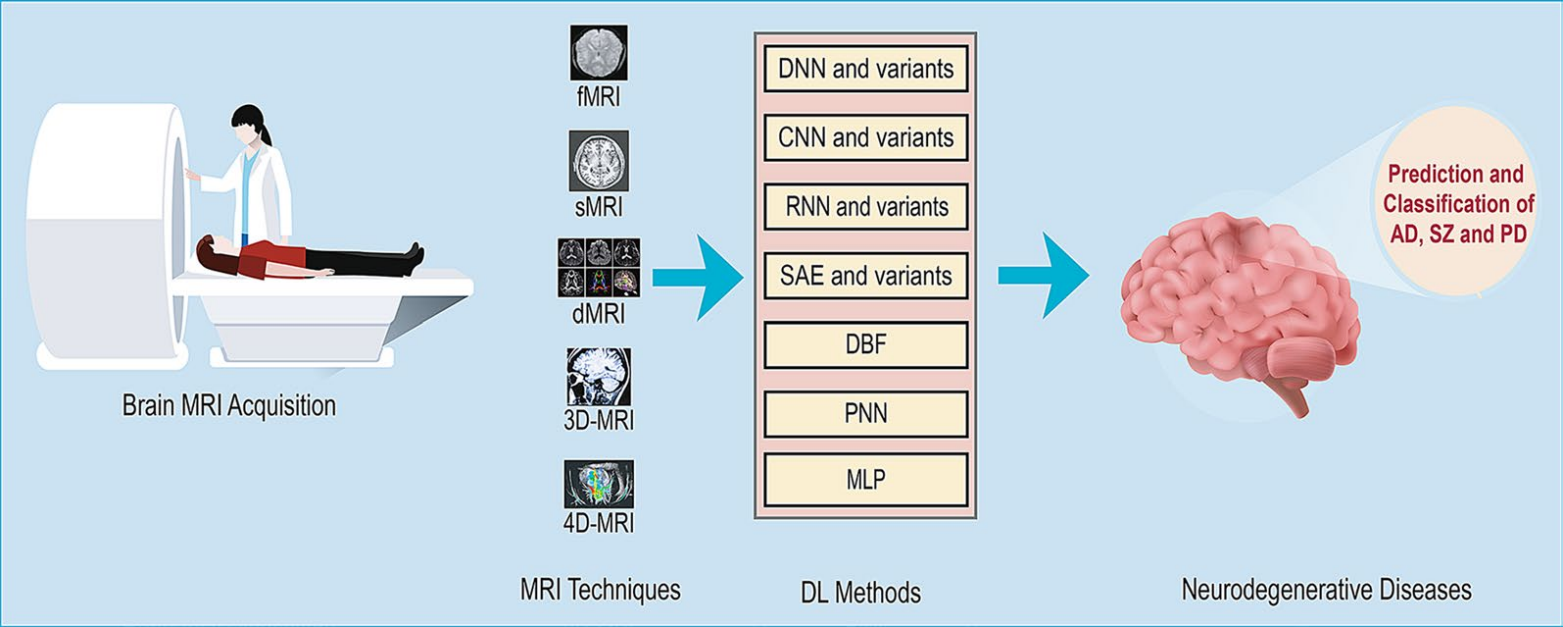
Overview of DL-based prediction and classification pipeline of neurodegenerative disease from different variants of MRI

DL is a sub-field of ML that can be used to build models which learn high-dimensional features from data. It has attracted huge attention in the last few years especially in image analysis. A number of DL architectures such as CNN, DNN, RNN, AE, Deep Belief Network (DBN), and Probabilistic Neural Network (PNN) have been reported in the literature. These structures possess the capability to classify various NLD with very high accuracy [12, 13]. Figure 2 shows an overview of the pipeline employed the acquisition and analysis of the MRI scans.

**Fig. 3:**
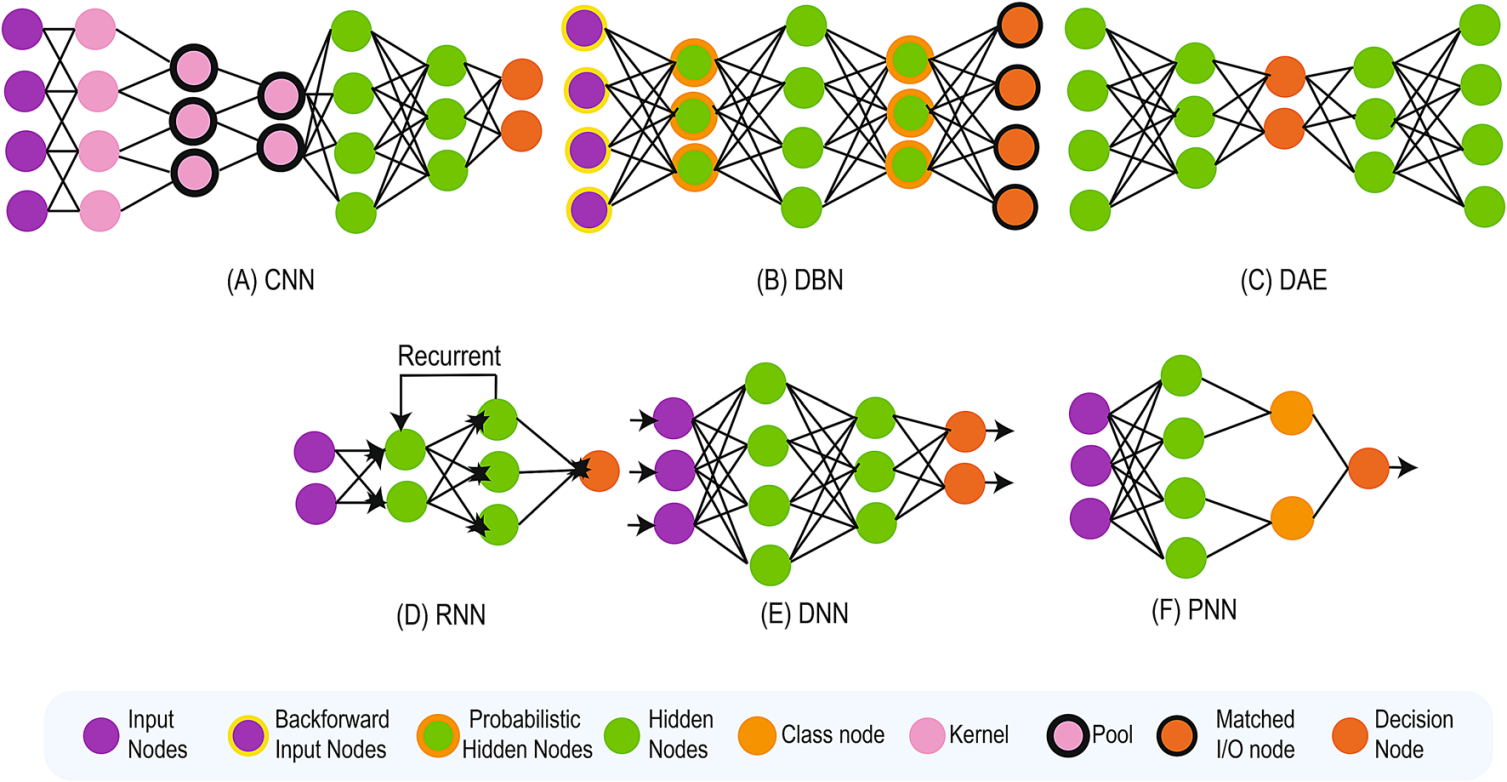
DL Architecture

### Convolutional neural network (CNN)

A CNN or also known as ConvNet (used alternatively in the text) (Fig. 3a) usually takes an input image, assigns learnable weights with biases to different aspects in the image subsequently differentiating one picture from the other. CNN uses convolution operation in place of simple matrix multiplication in at least one of their layers. It is mainly used in an unstructured dataset (e.g., image and video). 2D-convolutional kernels are used by 2D-CNN for the prediction of segmentation maps of a single slice. 2D-CNN can leverage features from only spatial dimensions (height and width). Since 2D-CNN takes only a single slice as input, they intrinsically fail to extract context information from adjacent slices. From practical perspective, voxel information from adjoining slices might contain enough information for the classification tasks. On the other hand, 3D-CNN can preserve temporal dimensions by predicting the volumetric patch of neuroimaging data [31]. Although 3D-CNN possesses the ability to anchorage interslice context information which leads to improved performance, but comes with a computational cost resulting in the increased number of parameters to be used by the 3D-CNNs. The various architecture of CNN are available (e.g., LeNet, AlexNet, VGGNet, GoogLeNet, ResNet, ZFNet, etc.) and can be used to build models for MRI analysis.

### Deep belief network (DBN)

DBN (Fig. 3b) is a mode of deep neural network (NN) which consists of a combined layer of a graphical model holding both directed and undirected edges. It consists of multiple layers of hidden units, in which each layer is linked with each other except the input units. DBN is mainly constituted of a stack of restricted Boltzmann machines (RBM) where each RBM layer needs to communicate with both the foregoing and successive layers. The nodes of any single layer do not communicate with each other distally. DBN are used to identify, group, and originate images, video clips, and motion-capture data. Its real-life application is Electroencephalography: An electrophysiological scanning method to document the electrical venture of the brain [32].

### Autoencoder (AE)

The AE (Fig. 3c) NN learns to facsimile its input to its output in an unsupervised manner. It has an internal (hidden) layer which is used to describe code to constitute the input. An AE consists of two main parts: an encoder which maps the input to the code, and a decoder which in turn maps the code to the remodeling of the original input. An AE has three common variants named as sparse AE, denoising AE, and contractive AE. The sparse AE architecture includes more hidden units than inputs yet only a limited number of the hidden units should be enabled to be active at any point of time forcing the model to retort to the unique statistical traits of the input data used for training. On the other hand, denoising AE takes a partially distorted input and is trained to reconstruct the original genuine input. And contractive AE gets to add an explicit regularizer in its objective function which gets to force the model to master a function that is resilient to a slight disparity of input values. An adaption of AE incorporating DL architecture is stacked AE (SAE) where multiple AE layers are stacked [33] to provide updated functionality by using a much detailed version of raw data with likely looking features to train a classifier with specified different contexts, subsequently finding better accuracy than training with raw data.

### Recurrent neural network (RNN)

RNN (Fig. 3d) is known as memory network which remembers the past and the decision it takes is influenced by what it has learned from the past. Thus an RNN can be considered as an architecture that consists of multiple copies of the same network where every other is passing a message to a successor. The principal and most important characteristic of RNN is its Hidden state. The function of the hidden state is to remember certain information about a sequence. The same parameters are used by every input as it has to perform the same task on all the inputs (hidden layers) for producing the output. This results in the reduction of the complexity parameters, unlike the other NNs. Application of RNN comprises in speech recognition, language modeling, translation, image captioning, etc.[34].

Long–Short Term Memory (LSTM) represents a variant of RNN. The main function of LSTM is to help to preserve the error which can be back-propagated through different time and layers. In LSTM information is stored outside the natural flow of the RNN in a gated cell. Like a computer’s memory, the cell is used for storing, writing to, and reading information. The cell itself decides about what to store, and when to allow read and update of the information via gates that tend to open and close when required.

### Deep neural network (DNN)

A NN having more than one hidden layer is generally referred to as DNN (Fig. 3e). In DNN every layer performs certain types of tasks embedding, collocating and ordering in a process. In deep feed-forward NN are also known as multi-layered perceptron (MLP) of neurons, information can travel only one-way (forward) with no feedback in the network. MLPs are capable of handling the complex non-linearly separable relations between input and output. In contrast, feedback NN has some kind of internal recurrence and hence feedback to a neuron or layer that has already received and processed that signal [35]. A substantial amount of annotated data is required for training a DNN. DNN can separate and extract internal features of millions of labeled images if trained with proper training algorithm.

### Probabilistic neural network (PNN)

PNN (Fig. 3f) is used for classification and pattern recognition tasks. In PNN the probability distribution function (PDF) is estimated using a Parzen window comprising a negative function. Then the probability of a new input data is computed using the PDF function. And finally, Bayes rule is applied to assign the new input data with the class that has the highest posterior probability. This method contributes to minimizing the misclassification of data [8].

**Table 1:** Data pre-processing techniques applied to MRI and fMRI images

Type	Ref.	Technique (applied methods)
Scaling	[36]	Image resize
	[8, 34, 37–39]	Image registration ( AR$^2$, DARTEL$^1$, LRg)
	[40–42]	Intensity non-uniformity correction (GW$^1$, B1-NU$^1$, N3$^1$)
	[42]	Distortion correction (PB$^1$)
	[43–45]	Bias correction/regularization (MICO$^1$)
	[46]	Contrast enhancement (CLAHE$^1$)
Correction	[31, 47–52]	Slice timing correction (HSI$^2$, FEAT$^2$, LSA$^2$, 6-PST$^2$)
	[31, 34, 40, 47–51, 53–55]	Motion correction (FSL-MCFLIRT$^2$, LSA$^2$, 6-PST$^2$)
Stripping and trimming	[39, 40, 44, 46, 53, 56, 57]	Skull stripping (NRBAC$^1$)
	[47]	Brain extraction (FSL-BET$^1$ $^2$)
	[36]	Trim edges
Normalization	[33, 34, 37, 39, 42, 47, 49, 51, 56, 58, 59]	Normalization (SPM $^{1,2}$, SD$^1$, ANTs$^1$, EPI$^2$)
	[36, 40]	Intensity normalization
	[31, 43, 45, 47, 50, 52, 54, 55]	Spatial normalization (ALT$^1$ $^2$, FSL-FLIRT$^2$, DARTEL$^2$)
	[32]	Z-score normalization
	[39]	Numerical normalization
Filtering	[46, 50, 51]	Basic filtering (GSF$^1$)
	[34]	Spatial filtering
	[34, 48, 49, 51]	Temporal filtering
	[8]	Weiner filtering
	[31, 47, 48, 53]	High-pass filtering
Smoothing	[45, 50, 58]	Basic smoothing (FWHM-GK$^2$)
	[31, 47–49, 51, 52, 54, 59]	Spatial smoothing (FWHM-GK$^2$)
Distinct techniques	[32]	Linear regression
	[49]	Linear detrend
	[31, 37, 43]	Modulation (JWF$^1$)
	[38, 44, 59]	Segmentation (LLL$^1$)
	[60]	Voxel-based morphometric
	[36]	Cortical reconstruction
	[34, 48]	Denoising (tCompCor$^2$)
	[46, 57]	Data augmentation

*FWHM-GK* Full-width half-maximum (FWHM) Gaussian kernel, *SPM *statistical parametric mapping, *SD* standardization, *ALT* affine linear transformation, *JWF* Jacobian of wrap field, *GSF* Gaussian smoothing filter, *FST* Free Surfer Tool, *ANTs* advanced normalization tools, *GW* Gradwarp, *B1-NU *B1-non-uniformity, *PB* phantom based, *FSL-BET* FMRIB Software Library-Brain Extraction Tool, *FSL-MCFLIRT* motion correction using FMRIB’s linear image registration tool, *FSL-FLIRT* FMRIB’s linear image registration tool, *FEAT* FMRI expert analysis tool, *CLAHE *contrast limited adaptive histogram equalization, *LLL* local label learning, *DARTEL* diffeomorphic anatomical registration through exponentiated Lie algebra, *LR* linear registration, *LSA* least square approach, *6-PST* 6 parameter spatial transformation, *EPI* echo planar imaging, *NRBAC* nonparametric region-based active contour, *MICO* multiplicative intrinsic component optimization

^1^MRI

^2^fMRI

## Data pre-processing techniques

The pre-processing step is important to enhance the quality of experimental data and preparing them for further statistical analysis. Different modalities of MRI scans acquired from several sources are susceptible to a broad range of noise including motion, average signal intensity, and spatial distortions which need to be removed from data to ensure correct analysis. There are a number of pre-processing techniques which have been applied on MRI and listed in Table 1.

### Scaling

Scaling is important to correct several issues in MRI scans including image resizing (IRE), image registration, resolution enhancement, correction, and so on.

*Image registration (IR)* Image registration is widely used in medical image analysis to align multiple images to verify the spatial correlation of anatomy across different images. Two types of registration algorithms are available: linear and non-linear. Linear registration (LRg) either exploits six-parametric rigid transformation (rotation and translation on x, y, and z axes) or 12-parametric affine transformation (rotation, translation, scaling, and shearing on x, y, and z axes) and global; whereas, non-linear registration can achieve a higher degree of elasticity which can model local deformation [8, 34, 61]. A toolbox “A Fast Diffeomorphic Registration Algorithm (DARTEL)” has also been reported to be useful in image registration [37].

*Intensity nonuniformity correction (INUC)* The smooth intensity variation in MRI scans caused by several factors such as non-uniform reception of coil sensitivity, radio frequency (RF) excitation field inhomogeneity, eddy currents driven by field gradients and electrodynamic interactions as in RF penetration, and standing wave effects as in intensity non-uniformity. In modern MRI scanners, these variations are tenuous enough that makes it difficult to detect. A solution to this approach comprises the usage of the convex accretion with an insistent method to enhance B1 uniformity in an anatomic region of interest (ROI) by differing the enormity and phase of every RF channel element separately [40–42]. A correction for nonlinearities in the gradients are applied by the scanner, called gradwarp. This correction tends to make the images spatially more accurate [41]. Large variance in the human brain’s response to substantial field inhomogeneity results in image distortion. Because the inhomogeneity field is slowly differing, it is a common practice to assume a smooth histogram. The N3 bias correction method is mostly used for that which is an iterative method that seeks the smooth multiplicative field maximizing the high-frequency content of the distribution of tissue intensity [41].

*Distortion correction (DC)* Functional MRI (fMRI) sequences generally pick up gradient echoes resulting in sensitivity to magnetic inhomogeneity (T2*) effects. Signal dropout near the skull base and spatial distortions are caused by this affecting anterior temporal and frontal lobes. These distortions can be reduced by applying available methods such as field mapping, unwarping, and phantom-based distortion correction [42].

*Bias correction and bias regularization (BC, BR)* A low-frequency but smooth bias field signal corrupts MRI images, specifically those which are produced by old MRI machines, for which several bias correction techniques might be applied as well [43–45].

*Contrast enhancement (CE)* A contrast enhancement method can be used to stop the clustering in histogram with the purpose of correcting the distribution. In [46], a CE method named CLAHE was used.

### Correction

Slice timing correction and motion correction are very important pre-processing steps applied to correct the slice-dependent delays of image slices and subject motion, respectively.

*Slice timing correction (STC)* Most fMRI studies do not acquire every slice in a volume at the same time. It signifies that the signal recorded from one slice might be offset in time by up to various seconds when compared to the other [48]. Thus, the time differences among the slices need to be accounted for. There have been two basic strategies for slice timing correction. The most commonly used method is data shifting where the recorded points are moved to contemplate their proper offset from the time of incitement. Interpolation of points is required for the method to fit the fixed, which is a TR-based timing grid using Hanning-windowed Sinc interpolation that produces some obscure and atrophy of the data as followed in [31]. Another strategy is model shifting, where the anticipated location of the hemodynamic response function (HRF) is differing [62]. Slice timing correction can also be executed by using FEAT module of FSL library [47]. Moreover, Least squares approach with 6 parameter spatial transformation is also used as a method for slice timing correction in [51].

*Motion correction (MC)* The largest source of error in fMRI studies is head motion which needs to be corrected during the acquisition of functional data. Trivial head movements also introduce unwanted variance in voxels and reduces the quality of data. Motion correction minimizes the impact of movement on image data by orienting the data to a reference time volume, application of which is found in [48, 54, 63]. Motion correction can also be performed by MCFLIRT module of FSL library [31, 47].

### Stripping/trimming

The skull stripping/brain extraction is a preliminary step in MRI analysis. A pre-processing step of trim edges (TE) has also been reported in the same context [36].

*Skull stripping (SST, BE)* Skull stripping/brain extraction is one of the most important pre-processing steps for eliminating non-brain tissues from brain MR images. It is important for many clinical applications and data analysis. To improve analysis speed and experimental accuracy of data, automated skull stripping is one of the helpful strategies. Brain extraction tool of FMRIB Software Library (FSL) has been used extensively for this purpose [47, 53]. Multiplicative intrinsic component optimization is applied for skull stripping in [44].

### Normalization (NM)

Normalization is a process of aligning and enclosing MRI data to a comprehensive anatomic template. Because of the difference in every person’s brain in terms of size and shape, normalization needs to be done to facilitate the comparison of one brain MRI to another in order to interpret them onto a common shape and size. Normalization tends to map the data acquired from discrete subject-space to a reference-space containing a template and a source image [64]. Usage of tools like Statistical Parametric Mapping (SPM) and Advanced Normalization Tools (ANTs) have been widely used in this context [33, 42]. Standardization has also been used simultaneously [33].

*Intensity normalization (IN)* Intensity normalization is used to reduce the intensity variation caused due to usage of different scanners or parameters for scanning different subjects or the same subject at disparate time [36, 40].

*Spatial normalization (SN)* spatial normalization warps the MRI scans to a similar stereotomical space, such that one MRI scan’s particular location matches other MRI scans from another subject [31, 43, 45, 52, 54, 55]. A common technique is affine linear transformation (ALT) [31, 37]. The FMRIB’s Linear Image Registration Tool or FLIRT module is also used for this purpose [47]. Moreover, diffeomorphic anatomical registration through exponentiated Lie algebra (DARTEL) procedure is used for spatial normalization in [50].

*Z-score normalization (ZN)* It is a strategy of normalizing data to avoid outlier issues by defining the divergence of sample data with respect to the mean of a distribution [32].

*Numerical normalization (NNM)* Numerical normalization is also found in the study which refers to the process of converting numerical values into a new range using a mathematical function. It contributes to make different experimental data values in different scales comparable resulting in their relationship to clearly stand out [64].

### Filtering (F)

It refers to the process of modifying or enhancing an image by emphasizing certain features or removing other features [50, 51]. Growing skull fracture or GSF has been reported in this study for reducing sharp pixel transitions between pixels [46, 58].

*Spatial filtering (SF)* Spatial filtering is that image enhancement technique which is used directly on pixels of an image where, value of the processed current pixel depends on both itself and adjacent pixels [34].

*Temporal filtering (TF)* Temporal filtering removes frequencies that are not of interest within the raw signal which substantially improves the signal-to-noise ratio (SNR) [34, 48, 49].

*Weiner filtering (WF)* One of the most prevalent signal-dependent noises in MRI scan is Rician noise which can be minimized using a popular filtering technique known as Weiner filter [8].

*High-pass filtering (HPF)* fMRI data tend to manifest low-frequency drifts at times, which is characterized by physiological noise and by physical (scanner-related) noise. These signal drifts might affect substantially statistical data analysis, if not removed. High-pass filters function comes into this context by cutting off frequencies below an acknowledged threshold which should be below the lowest frequency of interest [31, 47, 48, 53, 65].

### Smoothing (SM)

Smoothing refers to the process of reducing noise within an image which subsequently produces a less pixelated image [45, 58].

*Spatial smoothing (SS)* Spatial smoothing refers to the averaging of signals from adjoining voxels. It enhances the SNR but reduces spatial resolution, obscures the image, and smudges initiated areas into adjoining voxels. Since neighboring voxels are coordinated in their function and blood supply, the process can be difficult to perform. Within this context, the objective of spatial smoothing copes with functional anatomical variability which has not been compensated by spatial normalization (“warping”) thus, improving the SNR. Spatial smoothing is conducted with a spatially stationary Gaussian filter in which the user must ordain the kernel width in mm “full width half max” [31, 47, 48, 52–54]. This Gaussian kernel is a kernel which possesses the shape of a normal distribution curve [66].

### Distinct techniques

Apart from the techniques described above, some other pre-processing techniques have been reported in the study of the neurological disorders.

*Linear regression (LR)* Application of linear regression has been found in [32] to model the relationship between morphometric features and confounders.

*Linear detrend (LD)* Linear detrend is applied in those spectral estimation methods which are sensitive to the existence of linear trends being erratic for low frequencies [49].

*Modulation (MD)* A compensation or modulation is applied due to the contraction/enlargement of data of interest caused by the non-linear transformation. These data of interest comprise voxel of each registered grey matter image and is multiplied by the Jacobian of the warp field [31, 38].

*Segmentation (SG)* The process of segmentation is characterized by which the brain is partitioned into neurological sections following a specified template. It can be rather generalized for segmenting the brain into white matter, gray matter and cerebrospinal fluid. Segmentation is used for different purposes. In structural MRI (sMRI), this facilitates the normalization process. It also aids further analysis by the use of a specific segmentation as a mask and also can be used as the definition of a specific ROI [67]. However, local label learning has been used in [38].

*Voxel-based morphometric (VBM)* Voxel-based morphometric which actually uses statistics for identifying deviations in brain anatomy between groups of subjects has been used in [60].

*Cortical reconstruction (CR)* Cortical reconstruction is required for quantitative analysis of human brain structure [36].

*Denoising (DN)* MRI acquired from different sources are affected by noises. It results in loss of information associated with the image that might affect the quality of disease diagnosis or treatment. CompCor is a physiological noise correction method that exploits the noise ROI (e.g., white matter, ventricles, large vessels, and so on) to accurately predict the physiological fluctuations in gray matter regions. Noise ROI is defined using anatomical data to detect voxels that consist of either white matter or cerebrospinal fluid (CSF). A principal component analysis (PCA) is used to explain the variance in the time-series data derived from the noise ROI. Significant principal components (PCs) are fed as covariates in a general linear model (GLM) as an estimate of the physiological noise signal space. Voxels with largest temporal standard-deviation are known as tCompCor and have also been used in a number of studies [34, 68].

*Data augmentation (DA)* Data augmentation techniques are applied when the number of images for different classes become unbalanced [46, 57].

## Identification of neurological disorders

### Alzheimer’s disease

AD is characterized by escalating mental degradation that generally occurs in older age, due to deterioration of specific brain regions. However, a lot of research have been conducted to correctly discover the cause of this degeneration and automated ways to detect the patterns of degeneration from neuroimages.

**Table 2:** Summary of DL-based studies for prediction and classification of AD from MRI

Ref.	Reg.	DL Arch.	Pre-Proc.	Features	Dataset	Size	Accuracy
[33]	WB	SAE-3D, CNN	NM	CBF	ADNI	755 (AD, MCI, HC)	3-way 89.47%, AD vs. HC 95.39%, AD vs. MCI 86.84%, HC vs. MCI 92.11%
[31]		CNN	MC, STC, SS, HPF, SN, WMS, MD	SSIF	ADNI	52 AD$^3$, 92 HC$^3$, 211 AD$^1$, 91 HC$^1$	99.9%$^3$, $^{5\alpha }$, 98.84%$^1$, $^{5\alpha }$
[53]		CNN	MC, SST, HPF	SSIF	ADNI	28 AD, 15 NC	96.86%$^{5\alpha }$
[43]		CNN	SN, BC, MD	CBF	ADNI	33 AD, 22 LMCI, 49 MCI, 45 HC	98.88%
[42]		CNN	INUC, DC, NM		ADNI	193 AD, 151 HC	Class Score 95%$^{5\alpha }$
[69]		DNN		HPCV, CFV, LVV, ECT, MMSE	ADNI	60 AD, 60 HC, 60 cMCI 60 MCI	34.8%$^{10\alpha }$
[36]		3D-CNN	CR, TE, IRE, IN	3D CBF	ADNI	199 AD, 141 NC; 3D MRI AD 600 NC 598	98.74%
[56]		3D-CNN	SST, NM	CBF	ADNI	50 AD, 43 LMCI, 77 EMCI, 61 NC	
[8]		PNN	IR, WF	GLCM, SED	ADNI		85%
[70]		VAE, MLP	SG	Shape feature	ADNI	150 NC, 90 AD, 160 EMCI, 160 LMCI	NC-AD 84%, NC-EMCI 56%, NC-LMCI 59%. AD-EMCI 81%, AD-LMCI 57%, EMCI-LMCI 63%
[60]		DBN	VBM	VV 3611, MSD 24	OASIS	49 AD, 49 HC	MSD 0.7360$^{10\alpha }$, VV 0.9176$^{10\alpha }$
[47]		CNN	BE, MC, STC, IM, SS, THPF, NM, SN	CBF	ADNI	25 CN, 25 SMC, 25 EMCI, 25 LMCI, 13 MCI, 25 AD	CN 100%, SMC 96.85%, EMCI 97.38%, LMCI 97.43%, MCI 97.40%, AD 98.01%
[39]	BL	3D-CNN	NNM, BE, IR	4D features, clinical features	ADNI	192 AD, 184 HC, 181 pMCI, 228 sMCI	86%$^{5\alpha }$
[38]	HPC	CNN	IR, SG	HPC shape, texture, CBF	ADNI-1, ADNI- GO&2, AIBL	ADNI: 1711, AIBL: 435	
[71]		LSTM-RNN		LSTM-based features	ADNI-1, ADNI-GO&2	822 MCI	
[40]	CSA	SAE-DNN	MC, NUC, IN, SST, VL	310 Vol., CorTh, SAF, 5000 FDCM	ADNI, CAD- Dementia	171 CN, 232 MCI, 101 AD	Model-1 ADNI 56.6%$^{10\alpha }$, CAD-Dementia 51.4%$^{10\alpha }$ Model-2 ADNI 58%$^{10\alpha }$, CAD-Dementia 56.8%$^{10\alpha }$
[41]	MCS	CNN	INUC	CBF	ADNI	47 AD 34 NC	
[72]	SCS	CNN		CBF	OASIS	100 AD, 100 HC	VGG16: 92.3%$^{5\alpha }$, Inception-V4: 96.25%$^{5\alpha }$
[37]		CNN	NM, IR, MD	CBF	ADNI, Milan	ADNI: 294 PAD, 763 MCI, 352 HC Milan: 124 PAD, 50 MCI, 55 HC	ADNI: 99%$^{10\alpha }$, MILAN: 98%$^{10\alpha }$
[73]		CNN		CBF, 64	OASIS	416	80.25%
[46]	VB	CNN	SST, DA, CE, F	CBF	OASIS, MIRIAD	OASIS: 30 AD, 70 MCI, 316 HC MIRIAD: 46 MCI, 23 HC	0.8

*Ref* reference, *Reg* region, *DL Arch* deep learning architecture, *Pre-Proc* pre-processing technique used in the study, *WB* whole brain, *BL-brain lobes* HPC–hippocampus, *CSA* cortical surface area, *MCS* middle cross section, *SCS* single cross section, *SSIF* shift and scale-invariant features; Vol.-volume; CorTh-cortical thickness; SAF-surface area features; HPCV-hippocampal volumes; CFV-cerebrospinal fluid volume; LVV-lateral ventricle volume; ECT-entorhinal cortex thickness; MMSE-baseline scores of Mini-Mental State examination; $n\alpha$-n-fold cross-validation, *4DF* 4D features, *CF* clinical features, *GLCM* gray-level co-occurrence matrix, *SED* Sobel edge detector, *MSD*-maximal self-dissimilarity, *VV* voxel values

The authors in [33] have reported a DNN-based approach which consists of sparse AE and CNN by applying 3D convolutions on the whole MR images from subjects who are over 75 years of age. In this work, authors have obtained satisfactory classification results by using a 3-way classifier among healthy control (HC), AD, and mild cognitive impairment (MCI), i.e., HC vs. AD vs. MCI with an accuracy of 89.47%; and three binary classifiers (AD vs. HC, AD vs. MCI and MCI vs. HC). The approach captured local 3D patterns using the 3D-CNN which yielded better performance than 2D convolutions. Although in this experiment the convolutional layer has been pre-trained with an AE, it has not been fine-tuned to improve the classification performance.

The authors in [31] have differentiated HC and AD in older adults by extracting scale and shift-invariant low to high-level features using CNN. In this work, they proposed two pipelines of a workflow consisting of structural fMRI data with a classification accuracy of 99.9% and structural MRI data with a classification accuracy of 98.84%. In the first block of the pipelines, substantial pre-processing was performed to remove potential noise and distortion from the data. Next, a convolutional layer of CNN architecture which consisted of a set of learnable filters, serving as a shift and scale-invariant operator, extracted low- to mid-level features (also considered high-level features in GoogleNet). In the fMRI pipeline, both LeNet and GoogleNet had been implemented which were trained and tested by a massive number of images created from the 4D fMRI time series. The proposed model has been a highly accurate and reproducible. They have also contributed to characterizing multimodal MRI biomarkers. As for the limitation, this work has conducted experimentation concerning a fixed age-group limiting the possibilities to explore the patterns of different age groups.

Similarly, authors in [53] applied CNN (precisely, LeNet) to detect AD from HC. The pipeline consists of shift and scale-invariant features extraction by CNN followed by the LeNet model based on CNN model provided by Caffe DIGITS 0.2 from Nvidia to perform binary image classification. The classification accuracy obtained from this study was 96.86%.

Authors in [74] reported another framework by using hyperparameters from a very deep image classifier based on CNN to diagnose AD’s different stages. Here, the proposed model eliminates the necessity for the generation of a hand-crafted feature that transforms input to output by building a feature hierarchy from simple low-level features to complex high-level features. The proposed framework has utilized hyper-parameters from a very deep classifier, helping feature learning from small medical image datasets. This work is also the first one for detecting AD and classification utilizing DL methods on the OASIS dataset with an age group of 18–96 years. Classification accuracy achieved was 73.75% during a fivefold cross-validation. However, the authors have not validated the performance metric comparing with previous traditional methods.

Farooq et al. also used CNN for multiclass classification among AD, prodromal stages of AD, and HC [43]. They have proposed a CNN-based model where pre-processing of MRI images is first conducted to obtain grey matter images which are later passed to the CNN. In it, GoogLeNet and ResNet models have been used to train and test the CNN. The authors reported a 4% increase in classification accuracy compared to other methods selected from the literature. A very high 4-way (AD/MCI/LMCI/NC) accuracy of 98.8% and sensitivity of 97.9% for three classes (AD/MCI/NC). They have also contributed by not incorporating pre-trained features still enabling the network to predict the classes accurately.

Furthermore, Spasov et al. presented a parameter efficient 3D-CNN model to predict MCI to AD conversion along with the classification of AD and HC [39]. The model is based on 3D separable and grouped convolutions to extricate detailed descriptive features from sMRI. In this work, the authors have contributed in early identification of the MCI patients with a high risk of conversion to AD within 3 years. With a classification accuracy of 86% they also achieved sensitivity 87.5% and specificity 85.7% exploiting tenfold cross-validation. As the model contained parameter efficient layers, it restricted overfitting in exploiting the AD and HC data.

In another study, Böhle et al. classified AD and HC using layer-wise relevance propagation (LRP) of CNN on MRI data [42]. The authors compared LRP to guided backpropagation (GB), a gradient-based method, which revealed that LRP heatmaps can contribute to more accurate detection. It has also been reported that the LRP method is useful in a clinical context for a case-by-case analysis. As for the limitations, the heatmaps have no ground truth as they are only an approximation to what dominates the classifier in its decisions. Also, heatmaps just highlight voxels contributing to a certain decision of a classifier which does not allow making an assertion about the underlying causes. The study reported a class score of more than 75% for AD classification by applying fivefold cross-validation.

Basaia et al. also used CNN to distinguish among AD, MCI conversion to AD, and stable MCI based on a single cross-sectional brain MRI scan [37]. This study reported a successful overcome of the limitation of generalizing the findings across different centers, scanners, and neuroimaging protocols to attain both reproducibility and reliability of results. Certain drawbacks comprise that they could not exclude the presence of future conversion MCI among stable MCI patients. Also in order to improve the prediction capability of the model, it needs to be tested with cognitive, clinical, PET, and genetics biomarkers.

Ullah et al. came up with a CNN model to detect AD and Dementia from 3D MR image [73]. This model can be extended to generalize other disease detection as well. However, the accuracy of this experimentation could be enhanced with more training. They achieved an accuracy of 80.25% by applying cross-validation.

Amoroso et al. proposed a pure ML approach exploiting Random Forest for feature selection and a DNN for classification to early detection of AD [69]. This work was ranked third in the “International challenge for automated prediction of MCI from MRI data” which was hosted by the Kaggle platform and the work achieved an overall accuracy of 34.8 % by applying tenfold cross-validation over other participating teams. Although the classification results obtained by the authors with DNN got them to attain one of the most precise predictions in participant’s roaster, the multiclass classification accuracy is far from getting to competent results for clinical applicability.

A long–short term memory (LSTM)-based AE has been reported in [71] which consists of RNNs to learn compact and informative representation from longitudinal cognitive measures characterizes and facilitates the early prediction of MCI progression to AD. This work has achieved notable performance for predicting MCI subjects’ progression to AD using data within 1-year follow-up. Also, the proposed model built on data of later time points showed better performance than those which were built on data of earlier time points. On ADNI-1 they achieved a C-index value of 0.901 and 0.889 on ADNIGO-2.

Luo et al. provided an automatic AD detection algorithm using CNN on 3D brain MRI in which the 3D topology of the whole brain is considered [41]. The CNN architecture consists of three consecutive groups of processing layers, two fully connected layers, and a classification layer. In this work, the 3D topology of the brain has been considered as a whole in AD recognition which has resulted in an accurate recognition with a sensitivity value of 1 and specificity of 0.93.

Dolph et al. reported a model consisting of stacked AE (SAE) and DNN for multiclass classification that can learn complex non-linear atrophy patterns for classification of AD, MCI, and NC using both in-house and public-domain standardized CADDementia framework [40]. The authors produced two model specifications using blind datasets. Along with accuracy measurement, the authors also measured true positive fraction (TPF) to be 62.1% for AD, 54.5% for CN and 39.5% for MCI for the first model. Then for the second model, the authors achieved TPF of 64.1% for AD, 55.8% for CN and 51.6% for MCI. They have also contributed to include novel fractal-based texture fractal dimension co-occurrence matrix (FDCM) combining with well-known volumetric, cortical thickness, and surface area features for multiclass AD classification.

Bäckström et al. proposed a 3D-CNN for automatic learning of features and detecting AD on a pre-processed and fine-tuned large size MRI dataset using 3D-CNN [36]. This study has contributed to find the impact of hyper-parameter assortment on the performance of the proposed AD classifier with the impact of pre-processing, data partitioning, and dataset size. This work could be extended through subject-separated data partitioning tests.

A statistical feature gray-level co-occurrence matrix (GLCM)-based model exploiting PCA and finally PNN for training and classification has been proposed by Mathew et al. to classify AD, MCI, and NC [8]. This work achieved sensitivity measurement 86% of with specificity 83% and accuracy 85%. Proposed network architecture provides a better result than SVMs and KNN in terms of accuracy.

In [38], a DL model has been proposed using hippocampal magnetic resonance imaging data of 2146 subjects to predict MCI subjects’ progression to AD dementia in a time-to-event analysis setup. The proposed model is not sensitive to hippocampus segmentation requiring only a bounding box containing the hippocampus. This work went on to achieve a C-index of 0.762 for 6 to 78 months duration and C-index of 0.781 with 18 to 54 months duration. This model can be used in a cloud computing platform as well if containerized using Docker. This study focused on the hippocampus region, it is expected to obtain better performance if the DL method would have been applied to the whole-brain MRI data. Also, data at baseline were exploited in this study, whereas performance could be improved if longitudinal data were infused into the model.

Now, the authors of [56] have proposed a 3D-CNN architecture emphasizing to achieve better performance without incorporating feature extraction steps. Here two different approaches have been compared for MRI classification: the plain CNN and the residual NN. The proposed model’s performance was checked for the task of classifying MRI scans of subjects with AD, EMCI and LMCI, and NC. From the dataset, they tend to choose only the first image taken for every subject in order to eradicate possible information “leaks”. They have not provided the age group of the subjects. In terms of performance metric area under the curve (AUC), receiver operating characteristics (ROC) curves and accuracy have been evaluated using VoxCNN and ResNet. Of all the classifications presented in this paper, AD vs NC achieved the best result with AUC 0.88 ± 0.08 and acc 0.79 ± 0.08 using VoxCNN and AUC 0.87 ±0.07 with acc 0.80 ± 0.07 using ResNet In this study, there has been approached binary one-versus-one classification which showed better performance, an approach towards multiclass classification has not been tried.

In [72], the authors proposed a CNN-based architecture combined with transfer learning to separate AD patients from the HC group. Two architectures VGG16 and InceptionV4 have been exploited to carry out this task. The main emphasis has been put into building the architecture using a small training set through image entropy. Fivefold cross-validation was applied to achieve accuracy. Default hyperparameter values chosen for the models provide better results, whereas the hyperparameter search method could result in further improvement.

The authors in [70] have proposed a deep variational SAE-based approach which tends to learn latent feature (i.e., spectral feature) representation from the low-level features finally training an MLP for classification purposes consisting of six binary classification problems: AD vs. NC, NC vs. EMCI, NC vs. LMCI, AD vs. EMCI, AD vs. LMCI, and EMCI vs. LMCI. A softmax classifier has been applied to conduct the classification.

Furthermore, in [60] another approach based on DBN architecture has been proposed. Voxel-based morphometric (VBM) approach has been used for feature extraction here. Overall DBN has been depicted as a superior architecture in high-dimensional data classification. For mean-squared displacement or MSD feature vector sensitivity, specificity and accuracy found were 0.7122, 0.7601, and 0.7360. Also for VV-based feature vector, it provided better performance for sensitivity, specificity, and accuracy with 0.9059, 0.9296, and 0.9176.

However, a recent attempt of multiclass classification of 6 AD stages have been found in [47]. In this work, CNN-based Resnet-18 architecture with transfer learning has been used on rs-fMRI for training and evaluation purposes incorporating a good amount of pre-processing; whereas according to the literature presented in this work, the previous works on rs-fMRI were mainly based on LetNet, GoogleNet and Alexnet architectures. Several performance metrics have been used as well to evaluate their proposed model which are precision, recall, f1-measure, AUC, and ROC curves. Improved results in terms of accuracy have been found for 6 AD stages classification depicted in Table 2.

Consecutively another CNN-based model has been proposed in recent times in [46]. T1-weighted volumetric MR images have been used to diagnose AD and MCI. Two datasets were used for this work, where the OASIS dataset has been used for training purpose replicating the MIRIAD dataset for testing purposes. In this work, the authors have contributed by not applying particular age limitation in AD samples annotation which subsequently resulted in a stimulating prediction problem owing to a vast range of age distribution. By incorporating SST in pre-processing and using CNN-based features of the input images this proposed model attained accuracy values around 0.8 for diagnosis of both AD and MCI.

Table 2 presents a summary of mentioned DL applications for AD including the type of MRI, brain region and network involved, type of feature used with feature count, pre-processing technique used, dataset and number of participants from the dataset, validation scheme and performance accuracy found for all reviewed papers.

### Parkinson’s disease

Parkinson’s disease or PD is a neurodegenerative disorder that affects voluntary movements. As identification of PD as well as its underlying causes is very crucial to devise treatment strategy, DL has also been applied to detect it from neuroimages. A number of studies have been reported to serve that purpose. Table 3 presents a summary of these studies which employed DL applications for PD including the type of MRI, brain region and network involved, type of feature used with feature count, pre-processing technique used, dataset and number of participants from the dataset, validation scheme and performance accuracy found for all reviewed papers. Kollias et al. proposed a DNN architecture including CNN deriving rich internal depiction from input data and bidirectional-LSTM/gated recurrent units (GRU RNNs)-based RNN to analyze time progression of the inputs for delivering the final predictions [75]. A combined supervised and unsupervised learning methodology has been developed here exploiting ResNet and ReLU architectures. They have contributed to the creation of a new database that has been used for training, evaluating, and validating the proposed systems. Shinde et al. proposed to differentiate PD from HC by employing a fully automated CNN with discriminative localization architecture for creating prognostic and diagnostic biomarkers of PD from Neuro-melanin sensitive MRI or NMS-MRI [76]. For this work, data have been collected from the Department of Neurology, National Institute of Mental Health and Neuro sciences (NIMHANS) which consist of MR imaging, demographic and clinical details such as gender, age at presentation, age at onset of motor symptoms, disease duration, etc., data of PD patients, atypical parkinsonian syndromes (APS) patients, multiple system atrophy (MSA) patients and progressive supranuclear palsy along with some HC as well [76]. The authors were able to capture the subtle changes in PD in the substantia nigra pars compacta (SNc) using selected features from the NMS-MRI. Although the proposed method shows satisfactory performance exploiting a small sample size, larger sample size is required for improved efficacy of the method. On the other hand, Kollia et al. proposed a convolutional-RNN architecture for PD prediction through the extraction of latent variable information from trained DNN using both MRI data [77]. In this work, the authors presented a DNN retraining procedure, which allowed retaining the knowledge provided by previously extracted, annotated, and clustered latent variables. Later on, the information provided by those clustered latent variables were used to develop a domain adaptation approach. It tends to improve the performance of the DNN architecture even if presented with less input.

Using sMRI, dopamine transporter (DAT) scan data, age, and gender information, Pereira et al. [45] proposed a novel model to detect PD patients via CNN. The authors observed that pattern changes in the basal ganglia and the mesencephalon can be considered as a dominating feature for the detection of PD from HC and scans without evidence for dopaminergic deficit (SWEDD).

Esmaeilzadeh et al. used a 3D-CNN incorporating a voxel-based approach for brain image segmentation extracting data augmentation techniques to expand the training set size to classify PD and HC [57].

Moreover, Sivaranjini et al. contributed in analyzing T2-weighted MRI scans to classify between HC and PD by applying deep CNN architecture AlexNet [58]. In this study, sensitivity and specificity with values of 89.30% and 88.40% were also evaluated with a classification accuracy of 88.90%.

**Table 3:** Summary of DL-based studies for prediction and classification of PD from [s]-MRI

Ref.	Regions	DL Tech.	Pre-Proc.	Feature	Dataset	Size	Accuracy
[75]	Axial	CNN-RNN	–	CBFd	NTUA	55 PD, 23 PD Synd	98%
[57]	Sagittal, coronal, axial planes	3D-CNN	SST, DA	CNN based, age, sex	PPMI	452 PD, 204 HC	100%
[76]	Mild brain	CNN		CBF	NIMHANS	45 PD, 20 APS, 35 HC	80%$^{5\alpha }$
[77]	Lentiform nucleus	CNN-RNN		CNN based	NTUA	66176	98%
[58]	Whole brain	CNN	NM, F, SM	CBF	PPMI	100 PD, 82 HC	88.9%
[45]	Basal ganglia, mesencephalon	CNN	AC, BR, SN, SM	CNN based	PPMI		Control vs PD 94.5-96%, PD vs SWEDD 88.7%

* Pre-Proc.* pre-processing, *Synd* syndrome, $n\alpha$– *n* fold cross-validation, *AC* alignment correction, *SWEDD* scans without evidence for dopaminergic deficit, *CBF* CNN-based features

### Schizophrenia

Schizophrenia or SZ is a major psychiatric disorder related to structural and functional brain anomalies that gradually ended up with impairments in cognition, emotion, and behavior. In recent years, many researchers have contributed to develop automated tools and techniques for the initial diagnosis of SZ using DL and MRI data. Table 4 provides a summary of DL techniques applied in prediction and classification of SZ.

Qureshi et al. have proposed 3D-CNN-based DL classification to distinguish patients with SZ and HC [48]. The rs-FMRI data collected from the Center for Biomedical Research Excellence (COBRE) dataset was first preprocessed using FMRIB Software Library (FSL) version 6.0. Afterward, the group independent component analysis (ICA)-based connectivity measures (maximum 30 independent components) were acquired using the enhanced version of FSL. The features are further normalized and thresholded to semi-automatically separate the noise and artifacts. Finally, 3D-CNN classification was applied and 98.09±1.01% tenfold cross-validated classification accuracy was achieved. But, the specific feature was not selected from ICA and hence contribution-based ranking of features was missing in this study. Moreover, a quite similar approach was found in [54] but the authors have applied 2D-CNN instead of 3D-CNN as a classifier and functionally informative slices are selected and labeled before classification. Data are preprocessed through motion correction and spatial normalization. The study has classified both slice level and subject level. For slice-level classification, the proposed method demonstrated an average accuracy of 72.65% in the default mode network (DMN) and 78.34% in the auditory cortex (AUD). The study also shows better specificity in the DMN (80.75%) and higher sensitivity (79.11%) and specificity (77.25%) in the AUD. In short, according to the proposed work, 2D-CNN improved the accuracy of classification by reducing training parameters compared to 3D CNN.

A large portion of the reported approaches have applied DNN-based classification technique to diagnose SZ [35, 55, 78, 79]. Srinivasagopalan et al. claim that DL can be a paradigm shift for SZ diagnosis [78]. Their main finding was the ranking of features according to significance is important in detecting SZ in patients. Data were preprocessed using the ICA to achieve independent components and spatio-temporal regression to mitigate low bias or high variance. Recursive feature elimination and random forests were used to determine the importance of different features and to decide threshold cut-off for feature elimination. For classification, the authors implemented a simple three-layer DNN architecture and achieved a classification accuracy of nearly 94%. However, the training dataset used in this model was very small compared to the test dataset that may negatively affect the performance of the classifier. Matsubara et al. [79] proposed a deep neural generative model (DGM) implementing DNN for diagnosing psychiatric disorders from rs-fMRI data. The dataset used was already preprocessed with time-slice adjustment, rigid body rotation to correct for displacement, and spatial normalization. DGM evaluates the contribution weight of different brain regions to the diagnosis using Bayes’ rule. The proposed DGM implemented a ROI-wise feature and showed an acceptable performance (accuracy 76.6%, sensitivity 84.9%, and specificity 58.5%). But, the method is only applicable to rs-fMRI data and not robust to correlated regions. Moreover, DNN and LRP was used in improving the classification accuracy of SZ patients [55]. Data were collected from seven different sites and preprocessed through motion correction, spatial normalization using the SPM8 software^1^. The preprocessed data were then slightly subsampled to voxels and afterward decomposed via PCA. DNN classifier was trained with 1/2 norm regularization (dropout and batch normalization) by using resting-state functional network connectivity (FNC) patterns as input. LRP serves as an explanatory layer that provides relevant details to identify mostly informative features. The study found that some functional connectivity between the frontal network and sub-cortical network exhibits the highest discriminating power in SZ detection. The cross-site prediction accuracy was 82% with sensitivity 86.68% and specificity 82.79%. Also, DNN-based multi-view models comprising deep canonical correlation analysis (DCCA), deep canonically correlated auto-encoders (DCCAE), and SVM with Gaussian kernel was used to determine SZ in [35]. It is noted that multimodal features (FNC, SBM) and ICA were mainly considered as parameters for DNN-based classification.

An MLP model was also applied to analyze normal and SZ subjects from multisite sMRI data in [59]. The sites were: the Johns Hopkins University, USA; the Maryland Psychiatric Research Center, USA; the Institute of Psychiatry, UK; and the Western Psychiatric Research Institute and Clinic at the University of Pittsburgh, USA. The work was based on the hypothesis that the NNs trained on synthetic data may provide better performance than trained on real data. To verify the hypothesis, the sMRI images were first normalized to Montreal Neurologic Institute (MNI) standard space followed by segmentation into gray matter, white matter, and cerebrospinal fluid maps. Finally, the resulting gray matter images were smoothed with an isotropic 8 mm full-width at half-maximum Gaussian filter and used as input for the data-driven simulator. ICA and random variable (RV) sampling method were used to reduce dimensionality and to generate synthetic samples, respectively. Through simulation, the best performance was achieved by the MLP classifier on synthetic sMRI with an average AUC 0.75. However, the range of the data size that can be fed to a simulator is not defined and the important brain region for classification is not identified for the study. Han et al. started their research for resolving whether resting-state functional connectivity can be used as a biomarker of clinical diagnosis of SZ [51]. A total of 70 subjects (39 early-stage SZs and 31 HCs) were recruited. rs-fMRI images were acquired and pre-processed using a least-squares approach to correct slice acquisition and head motion. Later on, the corrected images were normalized and filtered to get the functional connectivity features for feed-forward back propagation NN (FFBPN). The study found that rs-fMRI functional connectivity shows good potential classification capacity (accuracy: 79.3% , sensitivity: 87.4% specificity: 82.2%) and could be used as a biomarker of clinical diagnosis.

**Table 4:** Summery of DL-based studies for prediction and classification of SZ from MRI

Ref.	Regions	DL	Pre-Proc.	Feature (count)	Dataset	Size	Accuracy
[48]	VFN, CN, DMN	3D-CNN	MC, DN, STC, SS, TF, HPF	3D-ICA (15)	COBRE	72 SZs,74 HCs	98.09%$^{10\alpha }$
[54]	AUD, DMN	2D-CNN	MC, SN, SS	ICA(13)	Self	42 SZs,40 HCs	slice-level DMN-72.65%$^{5\alpha }$, AUD-78.34%$^{5\alpha }$, subject-level DMN-91.32%$^{5\alpha }$, AUD-98.75%$^{5\alpha }$
[78]	WB	DNN	ICA	FNC, SBM (10)	MRN	69 SZs, 75 HCs	94.4%
[79]	WB	DNN		ROI (116)	OpenfMRI	50 SZs, 49 BD, 122 HCs	76.6%$^{\alpha }$
[34]	WB	RNN	MC, DN, SF, TF, NM, LRg	SPF	FBIRN phase-II	87 SZs, 85 HCs	64%$^{10\alpha }$
[52]	WB	DNN	STC, SN, SS	FNC (116)	COBRE	72 SZs,74 HCs	95.4%$^{5\alpha }$
[35]	WB	DNN		FNC,SBM (410)	MLSP		
[32]	WB	DBN	LR, ZN	NMF	Multisite	143 SZs,83 HCs	73.6%$^{3\alpha }$
[50]	WB	SAE	STC, MC,SN, SM, F	VTS	COBRE	72 SZs,74 HCs	92%$^{10\alpha }$
[51]	Atlas	FFBPNN	STC, MC, TF, NM, SS	FNC (20)	Hospital	39 SZs,31 HCs	79.3%$^{10\alpha }$
[55]	WB	DNN, LRP	MC, SN	FNC, ICA (1225)	Multisite	558 SZs, 542 HCs	84.75%$^{10\alpha }$
[49]	Cor., Str., Cere.	DNN	MC, NM, STC, SS, LD, TF	FNC (116)	Multisite	474 SZs,607 HCs	$\approx$83%$^{10\alpha }$
[44]	Vent.	DBN	SST, BC, SG	SV, ROI	COBRE	72 SZs,76 HCs	ROI-83.3%$^{3\alpha }$, SV-90%$^{3\alpha }$
[80]	WB	MLP		ICA, RV	FBIRN	135 SZs,169 HCs	AUC- 0.85$^{8\alpha }$, SD-0.05
[59]	WB	MLP	NM, SG, SS		Multisite	198 SZs,191 HCs	AUC-0.75$^{10\alpha }$, SD-0.04

*WB* whole brain, *Cor.* cortical, *Str.* striatal, *Cere* cerebellar, *Vent.* ventricle, *MRN* mild research network, *VFN* visual frontal network, *AUD* auditory cortex, *CN* cerebellar network, *DMN* default mode network, $n\alpha =$n-fold cross-validation, *SPF* spatial feature, *NMF* neuro-morphometric features, *VTS* voxel time series, *SV* segmented ventricle, *Self* self-generated dataset

An attempt has been made to explore the performance of DBN in case of discriminating the normal and SZ subjects by taking ROI and morphometry data into consideration in [44] and [32], respectively. Latha et al. have pre-processed the COBRE dataset using skull stripping to remove the nonbrain tissue. Afterward ventricle region was segmented from the images using a multiplicative intrinsic component optimization method. The considered region was trained using DBN with learning method: stochastic gradient descent, adaptive gradient, and root-mean-square propagation [44]. The study achieved a high AUC value (0.899) for the segmented ventricle image with accuracy: 90%, sensitivity: 87.5% and specificity: 92.86%. On the other hand, multivariate analysis was done for visualizing the most affected brain regions in [32] with an error rate of 56.3% for classifying the first-episode SZ patients. Moreover, some current studies have employed autoencoder for functional connectivity feature extraction [49, 50, 52]. Thereafter, these trained features are applied to SVM classifier [50] or DNN classifier for automatic diagnosis of individuals with SZ [49, 52]. Apart from that Dakka et al. have successfully demonstrated the feasibility of R-CNN involving a 3D-CNN with LSTM units [34].

## Open-access datasets

### ADNI

Alzheimer’s disease neuroimaging initiative (ADNI) dataset includes demographic information, raw neuroimaging scan data, APOE genotype, CSF measurements, neuropsychological test scores, and diagnostic information [81]. ADNI is composed of ADNI-1 (it tends to develop biomarkers as denouement step for clinical trials), ADNI-GO (in this section biomarkers are examined in earlier stages of disease), ADNI-2 (biomarkers are developed as predictors of cognitive decline and as denouement also) and ADNI-3 (the usage of tau PET and functional imaging strategies are studied for clinical trials in this section). These subsections are again composed of the following type of data:

- ADNI-1 is composed of CN (cognitive normal), MCI (mild cognitive impairment), and AD (Alzheimer’s disease) data.
- ADNI-2 is composed of EMCI (early mild cognitive impairment) data.
- ADNI-GO is composed of CN, EMCI, AD, and LMCI (late mild cognitive impairment) data.
- Finally, ADNI-3 is composed of CN, MCI and AD data.

### OASIS

Open access series of imaging studies (OASIS) dataset includes longitudinal neuroimaging, clinical, cognitive, and biomarker data for normal aging and Alzheimer’s disease [82]. Currently, two sets of data are included in OASIS. One is cross-sectional which includes 416 subjects aged from 18 to 96, of whom 100 of them were clinically diagnosed with AD. The other one is a longitudinal section which comprises 150 subjects aged 60 to 96. In this section, the subjects were diagnosed with AD at certain points during their course of participation [83]. Kaggle dataset contains mild-to-moderate dementia dataset which is 72 subsets data taken from OASIS dataset.

### MIRIAD

Minimal Interval Resonance Imaging in Alzheimer’s Disease (MIRIAD) dataset consists of a series of longitudinal volumetric T1 MRI scans of 46 mild–moderate Alzheimer’s subjects and 23 controls. There is a total of 708 scans in this database which had been collected at intervals from 2 weeks to 2 years conducted by the same radiographer using the same scanner. It also accompanied information on gender, age and MMSE scores [46, 84].

### COBRE

The Center for Biomedical Research Excellence (COBRE) dataset is found to be prevalent in research regarding SZ. The dataset includes raw anatomical and functional MRI data from 147 subjects (72 SZ and 75 HC) of age range: 18 to 65. Phenotypic data (e.g., gender, age, handedness, and diagnostic information) of every participant are also available [85]. Many studies have utilized the OpenfMRI database which is a repository of neuroimaging data collected using a different form of MRI and EEG techniques since 2010. The dataset contains information about subject-level variables (e.g., gender, age, handedness, etc.), longitudinal and multi-session studies, structural, anatomical imaging data (e.g., T1, T2-weighted, MPRAGE, etc.), resting-state and task-based fMRI data, diffusion-weighted imaging data, physiological (e.g., pulse, respiration, etc.) monitoring output acquired during MRI experiments, behavioral data collected without MRI, and standardized metadata to describe the conditions and parameters of the experiment data [86]. It is a huge repository of 95 MRI datasets including 3372 subjects from different sources.

### FBIRN

The Function Biomedical Informatics Research Network (FBIRN) is an another SZ dataset which develops methods and tools for fMRI studies to assess the major sources of variation among the studies and to provide a distributed datasets for a clinical study. Multi-scanner brain imaging datasets are shared through the BIRN Data Repository (BDR). Moreover, the FBIRN Phase 1 dataset consists of 5 traveling healthy subjects (age: 20 to 29 years) with no history of psychiatric or neurological illness, each scanned with sMRI and fMRI on 10 different 1.5 to 4 T scanners. The FBIRN Phase 2 (87 SZ and 85 HC, age: 18 to 70 years) and Phase 3 datasets (186 HC, 176 SZ, age: 18-62) consist of subjects with SZ disorder along with healthy comparison subjects scanned at multiple sites [87]. Moreover, several studies have been found while reviewing that have collected data from multiple sites or different hospitals to validate their proposed model.

### Other datasets

*AIBL* Australian Imaging Biomarkers and Lifestyle Study of Ageing (AIBL) dataset contains MRI, PiB PET images, and clinical data of more than thousand participants having minimum age of 60 years [88]. These datasets can be used for detection of AD and MCI [83].

*NTUA* NTUA Parkinson dataset which consists of MRI, DaT Scans, and clinical data of 55 patients with PD and 23 subjects with PD-related syndromes. A total of over 42000 images are available for academic use [89].

*PPMI* Parkinson’s Progression Markers Initiative (PPMI) public domain database to detect bio markers of PD progression. The PPMI study dataset includes raw and processed MRI and single-photon emission computerized tomography (SPECT) images [90].

*Open fMRI* Open functional MRI (fMRI) database includes recorded MRI and EEG data while subjects were asked to perform tasks [86].

*FITBIR* Federal Interagency Traumatic Brain Injury Research (FITBIR) dataset includes MRI imaging datasets which can be employed for understanding the relation between traumatic brain and Alzheimer’s disease [91].

Table 5 contains a list of all the open-source datasets found during the study. The includes 95 MRI datasets taken from 9972 subjects [86].

**Table 5:** Open source datasets containing data of neurodegenerative disorders

Ref.	Dataset	Description
[81]	ADNI	Alzheimer’s Disease Neuroimaging Initiative (ADNI) contains MRI data for detecting and tracking AD
[85]	COBRE	The Center for Biomedical Research Excellence (COBRE) dataset includes MR data of 147 subject where 72 patients are suffering from schizophrenia
[92]	fastMRI	It gives 1.5/3T MR data from 6,970 fully sampled brain data of axial T1/T2 and FLAIR images
[87]	FBIRN	Function Biomedical Informatics Research Network (FBIRN) Phase 1 consists of 5 traveling healthy subjects (age: 20–29 years) each scanned with sMRI and fMRI on 10 different 1.5 to 4 T scanners, FBIRN Phase 2 (87 SZ and 85 HC, age: 18–70) and Phase 3 datasets (186 HC, 176 SZ, age: 18–62) consist of subjects with SZ or schizoaffective disorder along with HC scanned at multiple sites
[91]	FITBIR	Along with the other Imaging datasets, the Federal Interagency Traumatic Brain Injury Research (FITBIR) includes the open source datasets for AD
[93]	Kaggle	It contains mild-to-moderate dementia dataset which is 72 subsets data taken from Open Access Series of Imaging Studies (OASIS) dataset
[94]	NAMIC	National Alliance for Medical Image Computing (NAMIC) provides Brain Mutlimodality datasets
[89]	NTUA	It consists of MRI and DAT scan of those who are suffering from PD and also some NC
[82]	OASIS	OASIS-3, OASIS-2 and OASIS-1 contain 373 MRI data of 150 subjects, 434 MRI data of 416 subjects and 2168 MRI data of 1098 subjects, respectively
[95]	MIRIAD	The MIRIAD dataset contains volumetric MRI brain-scans of AD sufferers and HC elderly people. This database consists of 46 mild–moderate Alzheimer’s subjects and 23 controls
[86]	Open fMRI	It contains 95 MRI datasets of 3372 subjects and can be used detect AD and PD
[96]	PPMI	Parkinson’s Progression Markers Initiative (PPMI) database accommodates raw and processed MRI of parkinson’s progression data

## Performance analysis

All the referred studies included in this paper incorporate several aspects of work from AD prediction, MCI to AD conversion, multiclass AD classification, etc. Performance metrics have been evaluated in terms of finding accuracy, specificity, sensitivity, class score, ROC and AUC values, concordance index, etc.

First of all, accuracy is a metric that is used for evaluating classification models. Thus, classification accuracy provides the percentage of correct predictions. Then, scoring is also termed as a prediction. It is the process in which values are generated based on a trained ML model on the basis of giving some new input data. The created scores can represent predictions of future values.

In order to visualize the performance of the multiclass classification problem, AUC, ROC, etc., curves are used widely. Here, ROC is the probability curve on the other side, AUC is used to represent the degree or measure of separability. It reveals the capability of the model for distinguishing different classes. The higher is the value of AUC, the better the model is at predicting correctly. For referred studies here, the higher is the value of AUC, the better the model performs in distinguishing between patients having a disease and no disease [97]. Defining terms used in AUC and ROC are sensitivity and specificity.

Sensitivity is measured as the proportion of actual positive cases that have been predicted as positive (or true positive (TP)). Sensitivity is also termed as recall. Then there will also be found those proportion of actual positive cases, which would be predicted incorrectly as negative (can also be termed as False Negative (FN)). For higher value of sensitivity rate of TP will be higher contrasting lower value of FN. Similarly, for lower value of sensitivity rate of TP will be lower contrasting higher value of FN. For the referred studies, models with high sensitivity show better performance [98, 99].

Specificity is measured as the proportion of actual negative cases that has been predicted as the negative (or true negative (TN)). Similarly, there will be another proportion of actual negative cases that has been predicted as positive (or false positive (FP)). For higher value of specificity rate of TN will be higher contrasting lower value of FP. Similarly, for lower value of specificity rate of TN will be lower contrasting higher value of FP [99].

Then, the concordance index or c-index is a metric that is most commonly used to evaluate the predictions made by an algorithm specifically for survival models. Actual survival prediction is important in the scenario of neurodegenerative disease analysis. Survival analysis is conducted from the perspective that since, both the training data and the test data are subject to censoring, it was not possible to observe the exact time taken for an event regardless of how the data was split. The c-index is used to evaluate the accuracy of the ordering in the predicted time. It is interpreted as 0.5 for random predictions, 1.0 for perfect concordance, and 0.0 for perfect anti-concordance. Generally, the concordance index for fitted models ranged between 0.55 and 0.7 owing to the presence of noise in data [100].

Authors in [31] achieved the highest rs-fMRI classification accuracy of about 99.9% using CNN. But accuracy of 38.8% has been achieved which featured a scientific challenge placing third over 19 participating teams to classify AD which is comparatively lower than all other [69].

**Fig. 4:**
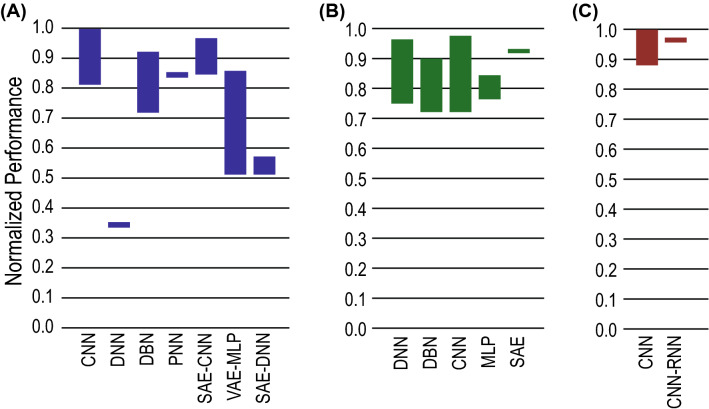
Performance comparison of application of various DLs in detecting neurological disorders from MRI datasets. The normalized performance for **a** Alzheimer’s disease, **b** schizophrenia and **c** Parkinson’s disease detection shows which method works well on which type of disease. The height of the bars denote the range of performance values reported in the literature

Highest accuracy 98.09$\%$ of schizophrenia detection has been observed in [48], which have employed 3D-CNN-based classification. Above 90$\%$ accuracy is shown in [78] and [44]. The other studies perceived the accuracy ranges from 70%-80$\%$.

By using 3D-CNN [57] achieved 100% accuracy on the validation and test sets for PD diagnosis. At the same time, the study in [76] discriminated PD from typical parkinsonian syndromes having 85.7% test accuracy.

Pereira et al. [45] found an accuracy of 96% using age as additional feature and CNN classifier for differentiating HC and PD, while the accuracy has dropped to 88.7% in classifying PD and SWEDD.

After analyzing the literature on AD, PD and SZ using DL some observations can be made based on the reported studies. The application of CNN is the most prevalent one in AD and PD detection. At the same time, ADNI has been the most used and balanced database covered in the study for AD, while NUTA and PPMI have been the most popular for PD. But, in the case of SZ detection, the prevalence of DNN has been more prominent compared to other DL techniques. And, the most frequently used database found is COBRE for the works covered in this study. A summary of the observations achieved from this study in terms of DL methods and datasets is shown in Table 6.

Performance analysis of application of various DL methods in detecting neurological disorders from MRI datasets are shown in Fig. 4.

**Table 6:** Summery of various DL methods and datasets used in detecting NLD

NLD	DL methods	Datasets
AD	CNN	ADNI, OASIS
PD	CNN	NTUA, PPMI
SZ	DNN, CNN	COBRE, FBIRN

## Challenges and future perspective

DL-based frameworks for the prediction of NLD has become desirable with the massive improvement in the computing capabilities and better development of DL tools. Further research may be conducted for tuning the DL algorithms in improving inferences (i.e., similar training and test environment). Some of the challenges with corresponding future perspectives are outlined below:

- The supervised architecture is limited due to huge effort for creating label data, low scalability, and selection of appropriate bias levels. Unsupervised learning is not a usual option to be considered for image analysis. However, unsupervised architecture not only learns features from the dataset but also design a data-driven decision support system from these data. Thus unsupervised deep architecture can be used to solve medical imaging-related problems.
- Predicting NLD from imaging data in real-time is still an open challenge. However, stream processing has been introduced for processing high-volume data using a parallel computing algorithm.
- Designing a bias-free neuroimaging dataset is challenging as it is a patrimony of learning system which may create a computational artifact. The problem can be reduced by including a large dataset in the model and studying the relationship between extracted features and tune the parameters of the model.
- Adversarial noise can add with the neuroimages and may reduce the classification accuracy. Thus, the cancellation of adversarial errors is a challenge.
- DL algorithms present impact and accurate solutions for large datasets. However, the high-dimensional CNN such as 2D-CNN and 3D-CNN will provide high accuracy for the large and multimodal neuroimages. On the other hand, Generative Adversarial Networks (GAN) can generate synthetic neuroimages which may also be used along with CNN.
- The basis of achieving better results using DL techniques largely depends upon using large training datasets; unavailability of which is one of the biggest barriers in the application process DL in neuroimaging which also comes in as a result of preserving the privacy of patients. At the same time annotating those data is a big issue as well as requires expert intervention. Consequently, the dataset found for rare diseases are mostly unbalanced. A collaboration of the health industry, medical professionals, and data scientists are required to overcome this problem of dataset creation and annotation. At the same time, data augmentation techniques can be applied to overcome the problem of unbalanced data by modifying data volume and quality.
- Non-standardized acquisition of images causes difference in images pertaining to different datasets. This poses a big challenge in processing the neuroimages using DL. Application of transfer learning is recommended here to overcome this problem.
- A deep learning model is a black box that learns from data and can be used to simulate the process from where data was collected. These models are interpretable rather than explainable. The black-box, however, works badly when the model is used to predict with data which do not belong to the database. Rudin clarified that the method used to forecast a process in the explainable DL is too complex, highly recursive and difficult to understand [101]. Explanation therefore often does not provide adequate information to understand the DL mechanism. There is therefore often a debut between explainable DL and interpretable DL.

## Conclusion

Advancement in high-speed computing techniques and an unprecedented improvement in the development of novel DL-based techniques and models opens up unique opportunity to predict and manage a number of neurological disorders including Alzheimer’s disease, Parkinson’s disease and schizophrenia. In this paper, the most popular DL techniques have been explored in detecting those three leading neurological disorders from the MRI scan data. DL methods for the classification of neurological disorders found in the literature have been outlined. The pros, cons, and performance of these DL techniques for the neuroimaging data have been summarized. In the end, the open challenges and future trends have been discussed. Prime observation of this study included the maximum usage of CNN in the detection of Alzheimer’s disease and Parkinson’s disease. On the other hand, DNN has been used in greater prevalence for schizophrenia detection. At the same time, ADNI, COBRE, and PPMI datasets have been explored mostly for AD, PD and SZ, respectively.

## Data Availability

Data sharing is not applicable to this article as no new data were created or analysed in this study.

## Footnotes

1 https://www.fil.ion.ucl.ac.uk/spm/software/spm8/
